# Perspectives of pupils, parents, and teachers on mental health problems among Vietnamese secondary school pupils

**DOI:** 10.1186/1471-2458-13-1046

**Published:** 2013-11-06

**Authors:** Dat Tan Nguyen, Christine Dedding, Tam Thi Pham, Joske Bunders

**Affiliations:** 1 Faculty of Public Health, Can Tho University of Medicine and Pharmacy, Can Tho, Vietnam; 2 VU University, Amsterdam, The Netherlands

**Keywords:** Mental health, Depression, Anxiety, Stress, Academic pressure, Secondary school, Pupil, Youth, Vietnam

## Abstract

**Background:**

Secondary school can be a stressful period for adolescents, having to cope with many life changes. Very little research has been conducted on the mental health status of secondary school pupils in South East Asian countries, such as Vietnam.

The study aimed to explore perceptions of mental health status, risk factors for mental health problems and strategies to improve mental health among Vietnamese secondary school students.

**Methods:**

A qualitative design was used to address the main study question including: six in-depth interviews conducted with professionals (with two researchers, two psychiatrists, and two secondary school teachers) to learn about their experience of mental health problems among secondary school pupils; 13 focus group discussions (four with teachers, four with parents, and five with pupils); and 10 individual in-depth interviews with pupils who did not take part in the FGDs, to reflect on the collected data and to deepen the authors’ understanding. All interviews and FGDs were audio-taped, transcribed and analyzed for the identification of emerging issues using qualitative techniques of progressive coding, analytic memoing and ongoing comparison.

**Results:**

Our study confirms the need to pay attention to mental health of pupils in Vietnam. Depression, anxiety, stress, suicidal thoughts and suicide attempts were seen as major problems by all stakeholders. Mental health problems were mainly associated with academic pressure, resulting from an overloaded curriculum and pressure from teachers and parents to succeed. The study found that pupils’ mental health demands interventions at many levels, including at the level of government (Ministry of Education and Training), schools, communities, families and pupils themselves.

**Conclusions:**

Vietnamese secondary school pupils feel that their mental health status is poor, because of many risk factors in their learning and living environment. The need now is to investigate further to identify and apply strategies to improve students’ mental health.

## Background

Vietnam has changed rapidly over the past two decades; economic development and an open door policy of economic liberalization have stimulated both economic growth and social change [1]. A side effect of these changes is a transition in disease patterns. For large segments of the population, the main diseases are no longer the diseases of poverty, but increasingly diseases that are seen in wealthier societies [2-4]. However, the health problems related to a more prosperous lifestyle are not equally distributed across the country; there are increasing gaps between rich and poor, and between urban and rural areas [3,5]. There has been a rapidly growing public awareness of mental health problems, such as stress, anxiety, depression and suicide among adolescents [4,5]. Psychopathology and life stress may play major roles in suicidal behaviours, especially among rural adolescents. Some 17.6% of secondary school pupils in a study in the north and 34.0% of first year university students in another study in Cantho City in the south reported feelings of sadness and hopelessness every day for two weeks in the past 12 months [6]. Four studies reported a high rate (10%) of students who had considered attempting suicide in the past 12 months [7-10]. Prevalence rates of suicidal behaviour increased significantly with age, and female adolescents were more likely to report suicidal feelings than males. Other studies reported an association between smoking/substance abuse and emotional/behavioural problems among adolescents [11]. Those involved in physical fights and/or attacks had higher levels of alcohol problems and poor mental health [12].

Although these studies revealed a high prevalence of poor mental health among Vietnamese adolescents, there is very little organized health care for this age group as yet and also very little understanding of the health problems they are facing. Up to now, no studies have explored the perspectives of pupils, teachers and parents about mental health, or school-related factors contributing to mental health problems of Vietnamese youth. The aim of our study was to explore perceptions of mental health status, risk factors for mental health problems and strategies to improve mental health among Vietnamese secondary school students. The results can help to develop interventions that fit the needs of these pupils.

## Methods

### Methodological approach

An exploratory qualitative approach including individual interviews and focus group discussions.

### Time and study site

This study was conducted from September to October, 2010, in Cantho City, a city with a population of 1.2 million (2010) in southern Vietnam [13].

### Participants and methods

The main informants included teachers, pupils, parents, researchers, and psychiatrists. Data was collected by means of in-depth interviews and focus group discussions (FGDs). The data collection process followed three stages. Firstly, six in-depth interviews were conducted with professionals to learn about their experience of common mental health problems among secondary school pupils. Interviewees comprised two researchers at the Hanoi School of Public Health; two psychiatrists; and two high school teachers in Cantho City. Interviews lasted 40–60 minutes and were recorded. Subsequently, guidelines for in-depth interviews and focus group discussions for students, teachers and parents were developed. The guidelines were semi structured and changed several times, allowing for new information to be investigated in subsequent interviews.

Secondly, 13 FGDs (4 with teachers, 4 with parents, and 5 with students), with a purposive sample of 8 to 12 participants for each FGD, were conducted in three secondary schools: 1) Ly Tu Trong (LTT), a specialized school which recruits pupils with an excellent scholastic record, 2) Chau Van Liem (CVL), the largest and oldest secondary school, located in the inner city, and 3) Tran Dai Nghia (TDN), a new school located in a suburban area. The majority of the TDN students come from suburban and rural areas, and they have lower study grades. The focus groups were conducted by two Vietnamese facilitators to take notes and to make recordings, and lasted from 100–150 minutes.

The purposive sampling aimed to compose groups representing the range of gender, age, study results and urban/rural characteristics. Head teachers invited teachers of grade 10 to 12 students to participate in each FGD, according to the researcher’s instructions on wide representation with regard to gender, age, and teaching experience. Of the 46 teachers invited, 36 participated; the others said they had other appointments in their out-of-class time.

Pupils aged between 15 and 18 years old attending grades 10 to 12 were invited by head teachers on a day that pupils did not have classes all day. The pupils were selected on the basis of the researcher’s request for wide representation with regard to gender and to a range from high to low school performance. In the LTT specialized school, the 12 students in one FGD included three from each of the four specializations: mathematics and physics, English, biology and social sciences, from grades 10, 11 and 12. In the two bigger schools, CVL and TDN, the classes are divided into two groups: one with higher scores and one with lower scores in grade 10 entrance examinations. Therefore, in these two schools we had two FGD, one with lower scoring and one with higher scoring students. In each FGD, students also were considered for invitation with regard to gender, among those in grades 10 to 12. All invited students were selected from the class lists; of 60 who were invited, 55 participated. We do not know the reasons for the five not to participate.

Parents were invited by head teachers by letter or by telephone because teachers had closer contract with parents and they would be more likely to respond than if the researchers invited them. In each FGD, parents were invited based on wide representation with regard to having children in grades 10 to 12, with low and high school performance, different parental backgrounds and living locations (urban and suburban areas), and gender. Out of 48 parents invited, 34 agreed to participate. We do not know whether the others could not make time or did not wish to join the discussions.

Finally, after the FGD, in-depth interviews were conducted outside the schools with ten pupils who did not take part in the FGD, to reflect on the collected data and to deepen the authors’ understanding. In each school, the researcher invited one student of each grade from 10 to 12 to join as they left school. Not all students had time, however, due to extra classes or appointments with their parents; then another student was invited, from the same grade. The interviewees were informed about the purposes of the study and their parents gave permission for them to join the interview; all the interviews were conducted in quiet coffee shops outside the schools.

### Data analysis

Each interview and FGD was transcribed verbatim, and then translated from Vietnamese into English word by word. The translation was double checked by the researcher and a Dutch professor who can understand Vietnamese language and Vietnamese culture because of her long time working in Vietnam. All interviews were analyzed for the identification of emerging issues using qualitative techniques of progressive coding, analytic memoing and ongoing comparison. Data collection and analysis proceeded simultaneously to allow for new information to be investigated in subsequent interviews. Emerging themes informed the ethical analysis, suggested new issues for investigation and assisted in the identification of conceptual relationships.

Firstly, key passages in the transcripts were highlighted. Words and phrases were grouped by similarities to develop initial categories and recurring concepts. The next stage of the analysis, axial coding, involved refining this list by deleting or combining categories, followed by making connections between the categories and defining properties, for instance, context or preconditions. The final stage, selective coding, involved the identification of core categories. This was extended by deliberately seeking out negative cases which do not fit the theory and producing explanations for them.

### Ethics

This study was approved by the Scientific and Training Committee of the Cantho University of Medicine and Pharmacy. All respondents and parents of student respondents were informed about the study and given the option of participating; they were also informed that they could withdraw from the interview or FGD at any time if they did not wish to continue. Two parents stopped in the middle of the FGD because they had to leave early due to other duties.

## Results

In this section, the perspectives of pupils, teachers, and parents on mental health problems and causes, as well as first ideas for intervention strategies, are described. Most attention is paid to the pupils’ perspectives and the mental health problems they face. After describing the pupils’ perspectives, we consider whether parents and teachers recognize pupils’ stories.

### Study participants

The study participants included 36 teachers, 55 pupils, 34 parents, two researchers, and two psychiatrists who were invited to participate in the study. Most of those invited to participate agreed to do so. The male: female ratio was 40:60 for each group. The pupils ranged from 15 to 18 years of age. The teachers had 2–22 years’ experience of teaching at secondary schools.

### Pupils’ perspectives

#### Occurrence of mental health problems

According to the pupils, mental health was a large problem among them. At least a quarter of the 10–15 pupils in each FGD complained that they felt very stressed, anxious, and often worried. For example, one girl from CVL stated,

“I get a stomach ache, feel anxious, and find it difficult to concentrate when I have examinations or tests. My parents are over-anxious for me. I feel pressured and worry too much.”

Many students remembered other students with symptoms of depression. A group in an FGD concluded, “*About 10% to 20% students are often quite silent and seldom speak to anyone. They did not want to do anything in class even when they were asked to do something, and they did not care if they had good or bad results.”*

Poor mental health may lead to poor somatic health, as one boy illustrated:

“I felt dizzy when I sat or lay down and then stood up immediately. It was more severe when I had insomnia. I often have it because I worry about my examination and test results.”

Another girl added: “*I sometimes feel very sad and could not study anymore and do not want to have lunch or dinner anymore.”*

Though exact numbers were not available, in all three schools there have been several suicide attempts in recent years. Suicide is a sensitive topic for both family and school, but the number of reported suicides among pupils has apparently increased. Some pupils shared their personal story in the FGD. For example, one girl recounted:

“When my parents quarreled, my study declined because I kept thinking about my parents’ conflict and couldn’t focus on study. Sometimes I was scolded by my parents with no legitimate reasons and with very strong language. Some weeks ago, I felt very sad and had suicidal thoughts. I took sleeping pills and was admitted to hospital for about 1 week… I think if my parents keep acting like this, my study will not improve any more. Also, I often have insomnia because of worrying. Currently, I feel better and my parents pay more attention to me.”

In this case, parental conflict seemed to have caused the problems, but most pupils reported that academic pressure is the main cause for (thoughts of) suicide among students.

#### Factors contributing to poor mental health

According to the pupils, the following factors contribute to poor mental health 1) academic pressure, 2) indulging in pleasures like online gaming, and internet, tobacco smoking and substance use, and 3) love-life, especially homosexuality.

1. *Academic pressure*

The pupils consider academic pressure to be a huge problem. They pointed to an overloaded academic curriculum and to pressure from teachers, parents, peers, and themselves to do well. One girl commented:

Unfortunately, this is not an exceptional case, as teachers explained, but rather a common reality.

High expectation of their teachers increases the pressure, as one boy reported: “*The numbers of subjects and lessons are too many and the demands from teachers are too high. Therefore, students cannot satisfy teachers’ demands.”* A girl explained: *“Sometimes, we get high results from examinations but teachers are still not satisfied or think that high grades are a coincidence and not due to pupils’ skills or hard work.”* Other students confirmed that pressure from teachers distressed pupils, and could lead to despondency and loss of confidence. One girl explained: “*Because of fear, some students did not dare to look at the teacher’s face when they were reciting lessons in class. This fear impacts student’s ability.”* Pressure from teachers can have serious consequences, as described by one boy:

Along with concerns about the demands of teachers, parental pressure was raised as a common and serious problem; parents are very keen for their children to have a good career. One girl explained:

In Vietnam, parents are very focused on their children’s success and future career. One girl commented: *“Parents are less interested in care for their children but have high expectations of them. They require their children to study well like other children.”*

Pupils also experience pressure from their peers, as competition is fierce. A girl from LTT stated, *“If we have low marks, we have to leave the specialized class or to leave the group of excellent students, eligible for provincial and national competitions.”* Finally, academic pressure also comes from students themselves. A school girl from CVL, in answer to a question about why she easily gets upset and quarrels with other students, frankly said:

2. *Problems associated with pleasure seeking*

Although entertainment is part of normal life, most of the pupils thought that too much pleasure seeking could have a negative impact on students’ study and health [14]. Pleasure seeking behaviour that they thought could lead to problems included following media personalities, friendship, gaming, internet, and cigarette smoking. They also thought that these problems mostly occurred among pupils from rich families because they could use money for pleasure instead of needing it all for their study. According to the respondents, pupils who indulge in pleasures also often break school rules and show resistance to school regulations.

Students said that addiction to online computer games is high. According to one boy (CVL), “*The rate of online gaming is about 50% among boy pupils, (and this has) a negative impact on study results, due to spending too much time on it.”* Another girl remarked: “*Consequently, they did not spend enough time to study and received low results. Finally, they felt despondent and let things run their course.”* The main reason for game addiction is said to be academic pressure. Because of the overloaded academic curriculum, pupils have little time to reduce stress and gaming is an easy and quick way to relax.

Although smoking and substance abuse are prohibited in schools, some boys smoke. They usually smoke tobacco in the coffee shops close to schools and in the toilets. The students said that those who smoke tend to break school rules often and show resistance toward school and teachers.

3. *Love and Sex*

Some parents do not allow their children to be involved in relationships before finishing secondary school, especially girls, since they are afraid that their children will not focus on their studies. A girl from TDN:

According to the pupils, relationships may have negative consequences for scholastic success, and may lead to fighting, early sexual intercourse, unwanted pregnancy, and even suicide attempts. A girl from TDN School said: *“I know some friends who had boyfriends and started to have sexual intercourse. Some then had less study success. One girl became pregnant but her boyfriend’s family didn’t agree for them to marry. Then she had the baby, but the child died after birth.”*

Another girl, from CVL, stated, *“Some good students’ study results declined because of love problems and attempted suicide.”* A girl from TDN recounted: “*A girl student attempted suicide because her boyfriend didn’t want to marry her. He was still a secondary school pupil. This girl left her home after she was rescued from the suicide attempt.”*

Homosexuality was also mentioned in most interviews and FGDs with pupils, and was seen to be linked to mental health problems. Pupils considered that homosexual relations are appearing more frequently among young people, linked to greater personal freedom and a modern life style, far from the concepts of the feudal society of the past. Homosexuality was linked by pupils to early sexual intercourse, lost concentration on studies, and even suicide attempts. In a FGD among CVL students, a girl explained:

“There are 13 subjects in school, and I have to study the whole day (morning and afternoon) on even days (Monday, Wednesday, Friday) and half the day on the other days (Tuesday, Thursday, Saturday). In addition, I have extra lessons from private centres or from my teachers from 6:00 pm to 9:00 pm, and then I study by myself up to 11.00- 12.00 pm at night. During examinations, I have to get up especially early around 4:30 am to revise lessons.”

“Last year, a very good student in this school attempted suicide by jumping from the second floor. We think the reason was pressure from teachers. She was a specialized biology student and attended a provincial competition for two subjects - biology and using a calculator. However, her marks in class were not high. Teachers were not fair to her in class and often openly complained about her.”

“My parents put pressure on me, like I have to do better than other people. My parents also want me to be equal to or even better than my brother who is excellent at school work. When my results did not reach my parents’ expectations, they were very sad, angry and dissatisfied so that I feel very sad too.”

“Because of myself, I feel jealous of some friends whose study was less successful than mine before, but now they study better than me. I put high pressure on myself to be better than my friends. My study results used to be very good but now they are not as good as before. Therefore, I feel angry with myself. I don’t want to be inferior to my friends in any way.”

“My parents did not allow me to go out with friends and have a boyfriend because they were afraid that I would not concentrate on my studies. Sometimes I want to have a boyfriend to share things with and confide in, but I have to refuse many boys.”

“*It happens in both boy and girl students, but boys manifest it more clearly. I think the rate of boys who have homosexual orientation is about 5%. Some students’ families did not approve of their children’s sexual orientation, and some of those students attempted suicide.”*

##### 

**Parents’ perspectives** Parents agree that academic pressure is the main cause of mental health problems among students. They consider that Vietnamese youth has to study too much. According to the parents, the government has not developed good academic curricula. One male parent of an LTT pupil stated: *“Innovation is lacking in the policies on education and training, especially in academic curricula and teaching methods.”* Academic pressure seems to be higher for academically poorer pupils. *“Depression often happens in students who have low study results because they are often scolded and under pressure from teachers, friends, and parents.”* according to the father of a CVL pupil*.* But, as the mother of a CVL pupil explained: *“The level of anxiety* also *depends on factors in the student’s home environment, including economic conditions, parental problems, and unhappy family.”*

Pleasure seeking, put on the agenda by the students, was also recognized. A father remarked:

“Indulging in watching TV and playing games – if a pupil’s brain spends too much energy on playing games, they do not have enough energy to study. Consequently, children lose the focus on their study, sleep late, and get bored with studying.”

Although parents admitted that parental pressure contributed partly to student’s mental health problems, they put the main blame on teachers and the government. As one mother of a CVL pupil complained: “*Parents share the teaching role for children only at home; children spend half or more of their time in schools.”* In addition, parents are of the opinion that schools and teachers do not provide a happy learning environment for pupils. Schools are very strict, requiring that students listen to teachers but not promoting discussion with fellow students or working in groups, as the father of a CVL pupil explained:

“Pupils have little time and opportunity to communicate in class because they have to listen to teachers and they have no permission to talk in class during study time. They will be scolded by teachers if they talk in class.”

Some parents accused teachers of not being interested in the wellbeing of pupils. As one mother expressed it, *“In schools, teachers lack concern for their pupils. There is poor teaching capacity and schools do not create suitable recreation grounds including sport facilities for students to help reduce stress.”*

Parents also reported quarrels and fights between students during and after school, and within and between schools.

Like the students, parents related suicide to the high academic pressure. The father of a CVL student explained: *“About two months ago, a girl jumped from the second floor in the morning at school, after she had been scolded by her father. She was under academic pressure but no one around, including parents and teachers took care of her; teachers and parents put her under academic pressure. They still argue about the causes of her suicide. She had already tried to commit suicide once before, last year.”*

##### 

**Teachers’ perspectives** Teachers admit that academic pressure due to an overloaded academic curriculum is a main cause for mental health problems and unhealthy behaviours, such as addiction to games and internet and fighting among students. A female teacher from TDN said:

“Most pupils are severely stressed (even depressed) because of the heavy burden of knowledge required after the curriculum reform. Time in class is not enough to acquire the knowledge and students have to have private tuition to enter and stay in good schools and to pass university entrance examinations. Consequently, some students are often in a state of anxiety and do not know how to do simple work at home, then they do not totally integrate into society.”

Teachers are obliged to follow the regulations of the Ministry of Education and Training; the heavy curriculum puts high pressure on students, but also on teachers. This pressure on teachers is increased because they also receive low salaries. A female teacher explained:

“Teachers’ salaries are too low. They have to find other jobs or run extra classes at home to support themselves. If teachers’ salaries were high enough, they could pay more attention to teaching.”

Teachers also complained about the parents’ role in teaching their children manners and good behaviour, and about parents’ lack of concern. One middle aged female teacher from TDN complained: “*Nowadays, young people don’t know how to restrain their temper, they lack problem-solving skills, and feel heavy stress, and therefore they get angry easily and are quarrelsome, even fighting among themselves.*”

All of the teachers are familiar with this serious problem, as the painful example given by a male teacher illustrates:

“*We had a boy student who was very excellent at biology. He was chosen to enter the competition in biology for excellent students at provincial level. However, because of the pressure, he chose to commit suicide by cutting his wrists when the competition date was near.”*

##### 

**Thinking of solutions** There were few differences among pupils, parents and teachers in the proposed solutions to reduce pupils’ mental health problems and the results are combined in this section. The students would like reduced academic pressure, more attention from their family, more recreational activities supported by schools, and a friendlier learning environment. Parents would like teachers and schools to take more responsibility for the quality of teaching and to find better ways to teach their children. The teachers would like to see pressure on them reduced by lowering academic pressure and increasing salaries, and they also want the parents to take some responsibility for teaching children. Combining their ideas, the solutions outlined below were proposed to reduce students’ mental health problems. The following actors need to play a role in these solutions: 1) the Ministry, 2) schools, 3) local government and community 4) parents and 5) students themselves.

Firstly, it is thought that the Ministry of Education and Training needs to revise and renew the academic curricula. One boy at CVL School suggested key strategies:

“*Reduce the pressure from the academic curriculum and develop good methods to teach students. Choose the key subjects and invest enough time in them. Reduce less important subjects to leave time for students to study what they want.”*

In addition to reducing the curriculum load, the Ministry could play a role in establishing regulations relating to gaming, alcohol, and violence. It could promote new, modern ways of teaching. Raising the salary for teachers is also considered important, since it would reduce pressure on teachers to find extra sources of income and increase their commitment to their teaching in the schools.

Secondly, the schools could play a role in reducing academic pressure and creating a friendlier learning environment: “*Create a friendly environment between teachers and students in study and examinations.”* was the suggestion from a girl at LTT*.* Having a playground was considered important. The schools have a few sport facilities and music lessons, but not enough for the large numbers of students. The academic schedules also leave pupils little time to use the few facilities available. Some students suggested that schools should organize extra activities to strengthen social cohesion, provide social and life skills, reduce academic stress, increase friendship, and reduce discrimination and disunity among students. One boy student at CVL stated,

“Pupils need extra courses about sexual education, student relationships, students and family, adolescence and school health. These activities could improve the understanding among students and between students and teachers. That could reduce study pressure on pupils, and reduce discrimination among them. They would feel less isolated, especially those with sexual issues like homosexuality. Sexual education would increase pupils’ understanding of sexual health and help them to avoid doing the wrong thing. I also joined a group to promote education on sexual health. I think it is effective. I hope the school will create good conditions for pupils to develop.”

Pupils also proposed that schools should organize meetings to bring teachers, parents and pupils together, and organize short training courses for them, to provide knowledge about adolescent psychology: *“Schools should have a group to support pupils and recognize problems that arise.” A*ccording to the parent of a TDN pupil, “*Psychologists are needed to train students in life skills and problem-solving skills.”* Many parents and teachers remarked on the necessity to *“combine activities of school and family.”*

Thirdly, the community should take part in teaching and educating young people. *“Local government should control online gaming and ban alcohol and tobacco consumption of pupils, with strict laws on violation.”* according to one male parent of a TDN pupil*.* Collaboration among associations and unions, such as the Parents’ Association, Women’s Union and Youth Union, were also suggested to strengthen support for students, teachers and parents. A teacher from TDN stated: *“We should create a forum for students to exchange ideas and a psychological counseling group to support students when they have problems.”*

Fourth, parents should create a friendly environment so that children feel comfortable to share their cares, and they should let children decide some things for themselves, as one boy recommended:

“*I think we should tell our parents that they should not forbid children to go out with friends. Because if children only stay at home they will not understand what is happening outside and they will not adapt to the social environment.”*

Finally, pupils need to find ways to cope with stress. One boy in TDN noted:

“I often write a blog. I put my sadness and my emotion in the blog, and then my friends can share and give me advice. Then I can find solutions and think less about the bad things. I also reduce stress by listening to music and watching movies online.”

Seeking advice or help outside the family is also necessary because pupils cannot easily share their feelings with their parents. Schools do have Secretary Boards, Youth Unions and Parents’ Associations, and some schools have medical professionals that can be consulted, but these institutions do not yet function well. One reason is that pupils have little knowledge and skills on mental health and psychology. In addition, pupils with mental health problems may not recognize their own problems and may not seek help.

## Discussion

The most prominent findings of this qualitative descriptive study were the perceptions of the students that mental health problems occur frequently and that different stakeholders need to pay attention to the mental health of pupils. Depression, anxiety, stress, suicidal thoughts and suicide attempts were seen as major problems by all stakeholders and many painful examples were shared. Mental health problems were mainly associated with academic pressure, associated with an overloaded academic curriculum and pressure to perform well, from teachers, parents, and pupils, but also from the family environment and pupils’ recreational activities. The findings from this study also suggested that mental health problems among pupils should be addressed at many levels in Vietnamese society including government, school, community and family levels.

Our findings suggest that mental health problems in Vietnamese youth are a concern, which is consistent with the two previous studies of Vietnamese adolescents, which reported that approximately 9% had mental health difficulties [15], and of first year medical and pharmacology students in Ho Chi Minh, of whom about 40% had depressive symptoms [16]. Some commentators have posited that the cultural influence of collectivism compared to individualism, or the cultural influences of authority figures may be more repressive in Vietnam than in Western countries and that these factors are responsible for a high prevalence of mental health disorders [15,17]. Others argue that the negative effect of rapid social change may be to blame [18].

### Academic pressure associated with mental health problems

University entrance is based on the scores achieved in the entrance examination and prospective students require high scores to be admitted to universities. Securing a place in a public university is considered a major step towards a successful career, especially for those from rural areas or disadvantaged families. The pressure on the candidates is very high. It is estimated that nearly one million students take the exam annually but, on average, only 20% pass [19].

Given the highly competitive nature of the education system, many school pupils spend a great deal of time on extra classes after school and even during weekends and holidays. A study done in Ho Chi Minh City found that nearly 30% of secondary school pupils spent more than three hours a day on additional studies; 47% reported attending classes during weekends or holidays [20]. In the same study, two thirds of pupils were found to experience medium or high educational stress, based on the Educational Stress Scale for Adolescents [20]. It can be concluded that pressure to succeed in school education is intense in Vietnam, and appears to be increasing as society becomes more competitive.

Competitive stress can be a positive stimulus for achievement for young people but, if this stress is severe and prolonged, it can have a major impact on health and well-being. Educational pressure on young people is discussed widely in the media and society but much of this discussion is based on case studies and anecdotes, rather than systematic research, and there has been little research published from Vietnam regarding academic stress and youth mental health. A recent cross-sectional study revealed that educational stress was strongly associated with depression, anxiety, psychological distress, poor well-being and other behavioural factors [20]. These findings are also consistent with a study of Chinese adolescents showing that educational stress was the most predictive variable for depression, and had a strong association with suicide ideation among Chinese adolescents [21].

### School and social factors associated with mental health problems

This study has revealed potentially important school and social factors related to mental health problems among Vietnamese secondary school pupils. According to pupils’ perceptions, school-related factors, including school cohesion and school environment, had an influence not only on risk-taking behaviours but also on the mental health of pupils. This trend is consistent with data from Hanoi City and Ho Chi Minh City, Vietnam [7,10]. According to parents and teachers, society also plays an important role in pupils’ risk-taking behaviours because young people are exposed to internet shops, coffee shops, bars, clubs, online games and sex websites. Such shops are often located near schools and, at present, the Vietnamese government does not have appropriate legislation or control mechanisms to reduce those risks.

Violence among adolescents has been reported to be increasing, especially fighting and bullying among girl students outside of school. Increasing numbers of video clips of fighting among pupils have been posted on the internet by other pupils witnessing the fights. In Cantho City, the media reported 252 fights among school pupils in 2011 [22]. Although fighting was mentioned by most of the respondents, in this study we did not explore fighting between pupils in detail; more data would be needed to compare the situation in Cantho City with other regions in Vietnam and other countries.

### Family environment associated with mental health problems

Previous studies conducted in the USA show that the family environment can be a strong source of support for developing adolescents, when providing close relationships, strong parenting skills, good communication, and modeling positive behaviours [23,24]. A lack of family support and negative adult behaviours can have a negative impact on adolescent mental health. Our results are similar to those reported from Vietnam and Malaysia, in which an unhappy family environment, difficult family events like the death of a parent, regular conflict in the family, poor parental relationships, and economic difficulties were predictors of poor mental health and risk-taking behaviours [7-9,25].

### Pupils’ leisure activities

According to pupils and parents in this study, playing computer games or accessing the internet were activities undertaken by pupils to release stress. However, addiction to computer games or the internet had a negative impact on academic achievement and could raise the level of academic stress. Poor academic achievement and academic stress could be linked to poor mental health among pupils. That was the finding in a study of Turkish university students, where internet addiction was found to have a direct impact on depression, anxiety, and stress [26]. Research on internet addiction demonstrated that greater use of the internet is associated with social and psychological variables such as a decline in the size of social circles, depression, loneliness, lower self-esteem and life satisfaction, sensation seeking, poor mental health, and low family function [26,27]. However, there are no published papers about the impact of gaming and internet addiction on mental health among adolescents in Vietnam. Further study to look at this relationship needs to be undertaken.

Finally, love-life and sexuality were found to contribute to mental health problems in different ways. Young people may be anxious about their romantic and sexual relationships and parental prohibition of such relationships, but the processes involved have not been clearly explained in this or in previous studies. However, our results do reveal that love-life could be associated with poor mental health among Vietnamese secondary school students, especially for those who have had sexual intercourse with or without pregnancy. This is consistent with the findings of a study of US high school students in which depression was associated with sexual intercourse, intercourse before the age of 14, and non condom-use [28].

Homosexuality among young people appears to be increasing in Vietnam, also in school settings. One female student estimated that about 5% of students are homosexual but the basis of that estimate was not explored. However, in Vietnam, homosexuality is not yet a clear concept to everyone, because patterns and practices have changed rapidly in past decades. The Vietnamese language is still adapting to the 'new’ reality of homosexuality, especially in the countryside [29]. Therefore, a student with feelings of sexual attraction to the same sex might have poor mental health and even attempt suicide because homosexuals face stigmatization and discrimination in Vietnamese society. This link between the stigmatization and discrimination of homosexuals and poor mental health has been documented in studies in the USA and other countries [30], but yet not in Vietnam. Clinicians and staff of community-based agencies need to enhance their awareness of the possibilities of suicide attempts among homosexual and bisexual youth, undertake screening for risk and actively seek to reduce stress related to homosexuality [31].

### Strengths and limitations

The strengths of this study include the fact that different stakeholders contributed their perspectives, experiences and suggestions for improvement of mental health of pupils during the interviews and FGD and that the numbers of respondents were relatively large for a qualitative study. Data from multiple informants are often more reliable than data from single informants [15,32]. Although the large number of informants in each focus group, 8 to 12, could provide rich information with confirmation, the large group could also discourage participants from sharing information about sensitive subjects like mental health problems and suicide. The fact that this was a largely urban study may have given urban parents more opportunities to express their ideas in FGD, although the researchers tried to ensure that all parents were able to give comments. In addition, the use of purposive sampling by head teachers might affect the generalizability of the findings. The selected participants might have had more than average concern and responsibility for the topic. Then the study may have over- or under-estimated the importance of mental health problems among secondary school students. However, this was an exploratory study, providing background for a larger survey, which we expect to provide representative data among a larger number of respondents and as such, the insights gained are valuable.

## Conclusions

Vietnamese secondary school pupils feel that their mental health status is poor. Depression, anxiety, stress, suicidal thoughts and suicide attempts were perceived to be major problems. Academic pressure, including an overloaded academic curriculum and pressure from teachers, parents, and pupils, a stressful family environment, and excess attention to recreational activities were reported as the main factors associated with students’ poor mental health. Pupils, teachers, and parents should all take part in reducing academic pressure and enhancing mental health of students, collaborating with Vietnamese authorities, communities and schools to design effective interventions.

## Abbreviations

CTC: Cantho city; CTUMP: Cantho university of medicine and pharmacy; CVL: Chau Van Liem School; IDI: In depth interview; LTT: Ly Tu Trong School; TDN: Tran Dai Nghia School; FGD: Focus group discussion.

## Competing interests

The authors declare that they have no competing interests.

## Authors’ contributions

NTD, BJ and CD devised the idea of study. NTD coordinated the study and analyzed data with assistance of CD. NTD produced the first draft with assistance of CD. All authors revised and contributed to the final version of the manuscript. All authors read and approved the final manuscript.

## Pre-publication history

The pre-publication history for this paper can be accessed here:

http://www.biomedcentral.com/1471-2458/13/1046/prepub
